# Goal of Care Conversations in Motion: Using System Dynamics to Demonstrate a Communication Priming Intervention

**DOI:** 10.1093/geroni/igaf122.3340

**Published:** 2025-12-31

**Authors:** Christi Lero

**Affiliations:** Washington University in St. Louis, St. Louis, Missouri, United States

## Abstract

Health systems are challenged with how to best align treatment with patient values, particularly in the face of older adult patients and those with serious illness. As a result, tools have been developed to endeavor facilitation of goals-of-care conversations (GOCC), which is often seen as a clinical endpoint and research outcome during testing of such tools. However, rehospitalizations are a dynamic and somewhat usual feature of serious illness which remains uncaptured by current methodological approaches for GOCC development and testing. System dynamics is a method used to model systems and visualize interactions among its components via feedback loops, delays, and non-linear relationships. Using a recently published randomized clinical trial featuring a GOCC tool, system dynamics methods are applied using a causal loop diagram, demonstrating interactions of the tool within larger healthcare system and highlighting patterns that surface from its interactions. Three major insights emerged from this reconceptualization. First, there are multiple points at which a GOCC tool may interact with components of the healthcare system. Second, feedback loops reveal that GOCC are less likely to occur if they have already been completed and documented in the patient record. Third, GOCC are not ‘one and done.’ Rather, they need to be revisited at each change in condition or hospital admission. System dynamics offer insight into understanding complex interactions enabling us to predict how GOCC tools might behave within certain systems.

